# Cartilage turnover and intra-articular corticosteroid injections in knee osteoarthritis

**DOI:** 10.1007/s00296-018-3988-2

**Published:** 2018-02-02

**Authors:** Rainer Klocke, Kirsty Levasseur, George D. Kitas, Jacqueline P. Smith, George Hirsch

**Affiliations:** 1 Department of Rheumatology, Dudley Group NHS Foundation Trust, Russells Hall Hospital, Pensnett Road, Dudley, West Midlands DY1 2HQ UK; 2 Division of Musculoskeletal and Dermatological Sciences, School of Biological Sciences, University of Manchester, Oxford Road, Manchester, M13 9PL UK

**Keywords:** Knee osteoarthritis, Biomarker, Urine CTX-II, Intra-articular corticosteroid

## Abstract

Intra-articular corticosteroid injections (IACI) are commonly used interventions for pain relief in patients with knee osteoarthritis (OA). Biomarkers may be helpful in further elucidating how IACI exert their effect. The aim of this study is to look at the response of biomarkers of cartilage and bone metabolism after IACI in knee OA. Eighty subjects with symptomatic knee OA [45% male, mean age (SD) 64 (11) years] underwent routine knee joint injection with 40 mg triamcinolone acetonide and 4 ml 1% lignocaine. Knee pain (as pain subscale of WOMAC VAS) and biomarkers [C-telopeptides of type-II collagen (uCTX-II), and N-telopeptides of type-I collagen in urine; cartilage oligomeric matrix protein (COMP), hyaluronic acid, N-terminal propeptide of type-IIA collagen, and human cartilage glycoprotein-39 (YKL-40) in serum] were measured at baseline and 3 weeks after IACI. Radiographic severity of disease was evaluated using knee radiographs. Median uCTX-II, a cartilage degradation marker, was lower at 3 weeks post IACI compared with baseline: 306.3 and 349.9 ng/mmol, respectively (*p* < 0.01), which remained significant after Bonferroni correction. Apart from a weak trend of lower sCOMP post IACI (*p* = 0.089), other biomarkers showed no change after IACI. Both baseline uCTX-II values and the change in uCTX-II from baseline to 3 weeks post injection correlated with radiographic severity of joint space narrowing, but not osteophyte grade. No association between uCTX-II and pain was observed. This observational study suggests that IACI in knee OA may reduce cartilage degradation in the short term.

## Introduction

Knee osteoarthritis (OA) is one of the most common painful and disabling long-term conditions worldwide. Intra-articular corticosteroid injection (IACI) is a commonly used intervention in patients with knee OA. IACI have been shown to provide effective pain relief in the short term [1], although the predictors of response remain uncertain [2]. However, less is known about the biological effects of IACI on cartilage and bone.

A number of biomarkers in serum and urine have been investigated for their potential role in the diagnosis, assessment of disease burden, prognosis, and to a lesser extent, responsiveness to treatment in OA [3]. In general, such biomarkers relate to the processes of cartilage or bone turnover, or synovial inflammation, associated with OA [4].

C-telopeptide fragments of type II collagen (CTX-II) are created during articular cartilage breakdown and excreted in urine (uCTX-II) [5]. Studies have shown an association between the levels of uCTX-II and the incidence of knee OA, as well as the risk of hand and hip OA; and risk of progression in established and early knee OA cohorts [6]. Conversely, serum N-terminal propeptide of collagen IIA (sPIIANP) is thought to reflect collagen type II synthesis and altered concentrations have been linked to knee OA diagnosis and progression [3, 4, 6]. Urinary N-telopeptide (uNTX) is a marker of collagen type I degradation [4, 6], and as such a marker more relating to bone turnover. Serum cartilage oligomeric matric protein (sCOMP) is a non-collagenous protein resulting from cartilage breakdown, but is also present in other tissues such as tendon and synovial membrane. It has been found to be elevated in patients with knee osteoarthritis [5], and together with uCTXII, is considered one of the most consistent candidate biomarker associated with radiographic severity [7], radiographic incidence and progression of knee OA, and the incidence of painful radiographic knee OA [6]. YKL-40 (sYKL, also known as chitinase-3-like protein 1 or human cartilage glycoprotein-39) is produced in articular chondrocytes and synoviocytes and serum levels have been shown to correlate with the disease severity in OA [3, 4]. Hyaluronic acid (HA) is a glycosaminoglycan found in synovial fluid and serum HA (sHA) levels are thought to relate to the synovial inflammation in knee OA [3, 4]. Whilst fluid biomarker research in OA has expanded greatly over the recent years, it should be recognized that the evidence underpinning their potential role relies on correlation with structural imaging rather than direct arthroscopic or histopathological findings.

We chose the above biomarkers for their varied potential roles in the OA process and as promising candidate biomarkers on the basis of previous reports [3, 4] with the aim of looking at their response to IACI in patients with symptomatic knee OA, which has not been described in human disease before.

## Materials and methods

Subjects with a diagnosis of symptomatic, primary knee OA (according to ACR 1987 criteria [8]) referred for IACI of the knee by their attending clinician were recruited from rheumatology and orthopaedic clinics at this district general teaching hospital. Eligible participants were over 40 years of age and scored more than 20 mm on a 100-mm global knee pain visual analogue score. Subjects were excluded if they had IACI in the preceding 3 months; were on treatment with more than 7.5 mg prednisolone equivalents daily; or suffered with idiopathic pain syndromes such as fibromyalgia. Eighty consecutive consenting subjects, who underwent routine knee joint injection with 40 mg triamcinolone acetonide mixed with 4 ml 1% lignocaine, were assessed. Pain (as pain subscale of WOMAC [9], v3.1; VAS; standardized to 0–100 mm), urinary and serum biomarkers were measured at baseline and 3 weeks after IA injection. UCTX-II (ng) was measured in a morning urine sample using the Urine Cartilaps^®^ enzyme immunoassay (Immunodiagnostic Systems Ltd, Boldon, UK) and corrected for simultaneously tested urinary creatinine (mM). Other biomarker assays were sourced as follows: uNTX (Osteomark^®^, Alere Scarborough, ME 04074, USA), COMP (Abnova, 62126 Heidelberg, DE), HA (BlueGene Biotech, Shanghai, China), PIIANP (EMD Millipore, Billerica, MA 01821, USA) and YKL (Circulex™, Cyclex Co, Nagano, Japan). Maximum intra-assay and inter-assay coefficients of variation were 8.2 and 9.6%, respectively.

Radiographic severity of disease was evaluated according to a standard atlas [10] by two investigators, blinded to clinical information using recent (i.e., less than 6 months old) knee X-rays (anteroposterior, lateral and ‘sky-line’ patellar–femoral views). X-rays were scored for individual features of OA, i.e., joint space narrowing (JSN) and osteophyte formation (OP), graded from 0 to 3, i.e., absent to severe, with the highest grade at any compartment of the knee joint used for further analysis. The study had external ethical (REC references 11/WM/0102 and 12/YH/0457) and internal R&D approval. Informed consent was obtained from all the individuals participating in this study and the study was conducted in accordance with the ethical standards as laid down in the 1964 Declaration of Helsinki and its later amendments.

### Statistical analysis

Statistical analysis was performed using GraphPadPrism (version 4.03 for Microsoft, GraphPad Software, La Jolla, CA, USA). UCTX-II, uNTX, serum COMP, HA, PIIANP, YKL results at baseline and 3 weeks showed non-parametric distribution and were compared using the Wilcoxon signed-rank test. UCTX-II variance according to JSN grades of 0/1, 2 and 3 was calculated using the Kruskal–Wallis test. Correlations between biomarkers and other variables were assessed using the Pearson’s r (for continuous variables) and two-tailed, unpaired *t* test (for binary variables, eg gender), provided data distribution passed the D’Agostino and Pearson normality test. The significance level (*α*) was chosen to be < 0.05. Bonferroni’s method was used for correction of multiple testing of the six biomarkers.

## Results

Eighty subjects [45.0% male, mean age 64.1 years ± 10.9 SD, mean body mass index (BMI) 31.2 kg/m^2^ ± 5.8 SD] underwent IACI and had serum and urine samples taken at baseline and 3 weeks. The mean baseline WOMAC pain score was 52.0 ± 20.4 mm, median osteophyte grade 2 (IQR 1, 3) and median JSN grade 2 (IQR 1, 2). Median uCTX-II at 3 weeks post injection was lower when compared with baseline: 306.3 and 349.9 ng/mM creatinine, respectively (*p* = 0.0020, Wilcoxon signed-rank test, Table 1), which remained significant after Bonferroni correction. Apart from a weak trend for lower sCOMP post IACI (*p* = 0.089), no differences were seen for other biomarkers before and after IACI (Table 1). Both baseline uCTX-II values and the change in uCTX-II from baseline to 3 weeks post injection correlated positively with radiographic severity of joint space narrowing, but not osteophyte formation (Table 2).

**Table 1:** Biomarker levels at baseline and 3 weeks post IACI injection

	uCTX-II (ng/mM crea)	uNTX (nM/mM crea)	sCOMP (ng/ml)	sHA (ng/ml)	sPIIANP (ng/ml)	sYKL (µg/ml)
Baseline (median, IQR)	**349.9** (249.2, 556.1)	**32.7** (24.8, 46.1)	**738.6** (625.6, 1006)	**6.95** (4.89, 9.29)	**3.32** (2.53, 4.24)	**31.4** (18.2, 58.4)
3 weeks post injection (median, IQR)	**306.3** (177.5, 446.6)	**33.6** (21.6, 45.5)	**727.5** (561.1, 1020)	**6.99** (4.66, 9.24)	**3.51** (2.63, 4.41)	**34.3** (17.1, 57.0)
*p* value*	0.0020	0.31	0.089	0.52	0.52	0.67

*Crea* creatinine

*Wilcoxon matched pairs test. See “Materials and methods” for significance level (*p* < 0.05)

**Table 2:** Median uCTX-II values at baseline and median decrease in uCTX-II according to radiographic joint space narrowing (JSN) and osteophyte (OP) grade

JSN grade	0/1 (*n* = 33)	2 (*n* = 27)	3 (*n* = 14)	*p* value*
Baseline uCTX-II ng/mM crea (IQR)	**303.2** (144.7, 414.5)	**352.1** (273.6, 525.5)	**542.2** (386.8, 851.3)	0.005
Decrease in uCTX-II ng/mM crea (IQR)	**− 6.7** (**−** 44.9, 103.5)	**61.8** (**−** 20.3, 223.3)	**177.1** (**−** 19.5, 392.4)	0.048

OP grade	0/1 (*n* = 28)	2 (*n* = 24)	3 (*n* = 22)	*p* value*
Baseline uCTX-II ng/mM crea (IQR)	**313.5** (144.7, 402.8)	**357.0** (226.6, 575.2)	**453.7** (320.0, 556.1)	0.059
Decrease in uCTX-II ng/mM crea (IQR)	**5.6** (**−** 31.5, 103.5)	**84.0** (**−** 26.1, 219.2)	**69.2** (**−** 64.4, 250.3)	0.306

*Crea* creatinine

*Kruskal–Wallis test. See “Materials and methods” section for definition of radiographic grades and significance level (*p* < 0.05)

No significant association was found between baseline uCTX-II and baseline pain (*p* = 0.17, Pearson’s *r* = 0.16) or change in uCTX-II and change in pain post IACI (*p* = 0.82, Pearson’s *r* = 0.03). There was also no significant correlation between baseline uCTX-II and age (*p* = 0.40, Pearson’s *r* = 0.10), BMI (*p* = 0.11; Pearson’s *r* = 0.19) or gender (*p* = 0.49).

## Discussion

In this knee OA cohort, uCTX-II was significantly lower at 3 weeks following IACI compared to baseline, while other examined biomarkers that have been proposed to be relevant to the OA process, did not show significant change. Furthermore, baseline uCTX-II and change in uCTX-II correlated with radiographic joint space narrowing, but we did not see a relationship association between uCTX-II (baseline or change) and pain or radiographic osteophyte severity.

Urinary CTX-II are fragments of type II cartilage excreted into the urine. It is to date one of the best characterized potential biomarkers for OA in terms of diagnosis, severity and progression of the disease and are widely accepted to reflect cartilage degradation [3–5, 7]. Our data concurs with previous reports that showed higher uCTXII levels correlating with more severe radiographic disease in knee OA [7, 11]. A number of intervention studies have shown a reduction in uCTX-II in subjects with knee OA with treatments such as joint distraction [12] and intra-articular hyaluronic acid injections [13]. This has generally been interpreted as a signal of reduced cartilage degradation as a consequence of the intervention.

A change in uCTXII following IACI has been described in experimental OA studies [14] but, to the best of our knowledge, not in human disease. Due to the lack of a placebo control arm in our study, we cannot exclude the possibility of confounding factors contributing to our findings (e.g., regression to the mean) rather than the IACI intervention. The finding that both baseline uCTX-II and change in uCTX-II varied with radiographic severity of joint space narrowing of the injected knee joint would suggest that the change in uCTX-II is related to the index OA knee joint, rather than a result of interference with cartilage metabolism at other, co-incident OA sites. The weak trend of a corresponding change in sCOMP, as a non-collagenous hyaline cartilage constituent lends weak further support to the notion, that IACI may have an anti-catabolic or chondroprotective effect in knee OA at least in the short term. It must also be acknowledged that the concomitant injection of 4 ml 1% lignocaine alongside triamcinolone acetonide could have had an interfering effect on our findings. Intra-articular administration of local anaesthetic, especially with repeated injections or at higher concentrations are suspected to be chondrotoxic, although the effect of a single injection of local anaesthetic on cartilage is not known [15].

It would presently seem very improbable for any potential beneficial effect of IACI on cartilage degradation in human knee OA to exist beyond the short term. A previous randomized placebo-controlled double-blind study in knee OA patients, using intra-articular 40 mg triamcinolone acetonide every 3 months over 2 years, reported a neutral effect on serial radiographic joint space narrowing [16]. However, a recent trial of similar design and intervention using MRI to assess cartilage volume concluded a detrimental effect of IACI vs placebo on cartilage volume after 2 years [17]. Further independent, placebo-controlled studies beyond 3 weeks that examine fluid biomarkers (as well as serial MRI) in knee OA seem warranted before any firm conclusions about the effects of IACI on cartilage structure and biology in knee OA are possible.

In the clinical practice, IACI are administered to patients with knee osteoarthritis with the intention to improve pain in the short to medium term, a practice supported by systematic reviews [1]. Despite the association of uCTX-II with joint space narrowing of the index knee, we did not see any correlations between uCTX-II values (or the change in uCTX-II post IACI) and reported pain, and this concurs with reports by others [7]. This would suggest that the mechanisms by which IACI improves pain and reduces uCTX-II are different and not directly related.

In this study, there was no statistically significant change in biomarkers proposed to reflect bone turnover or synovial inflammation. This could be due to the unselected clinical spectrum of our cohort, as it is now recognized that OA represents a diverse group with predominant inflammatory, metabolic, structural, genetic or psychological profiles which is likely to require a stratified treatment approach [18].

The strength of our study is the investigation of several biomarkers, pertinent to the various pathways of the OA process, in a human cohort with knee OA, looking at IACI, as a clinically frequent intervention and in relationship to radiographic severity. The limitations are the lack of a placebo arm, lack of systematic collection of medication (anagesics, drugs affecting bone metabolism) and lack of biomarker estimations beyond 3 weeks post IACI. The concomitant injection of a local anaesthetic with corticosteroid may have acted as an interfering factor. Finally, our sample size may be too small to allow firm conclusions, considering the heterogeneous phenotypic nature of knee OA [18].

In conclusion, our study based on selected urinary and serum biomarkers suggest that IACI may reduce cartilage breakdown in knee OA in the short term. Further investigations into the short- to long-term effects of IACI on cartilage biology in knee OA appear warranted.
